# Preliminary Assessment of Quartz Sand Properties from Latvian Coastal Beaches for Potential Filtration Applications

**DOI:** 10.3390/ma19040809

**Published:** 2026-02-20

**Authors:** Yuri Dekhtyar, Renate Kalnina, Elizabete Skrebele, Hermanis Sorokins, Marks Gorohovs, Fricis Tenters

**Affiliations:** 1 Faculty of Civil and Mechanical Engineering, Riga Technical University, 6B Ķīpsalas Street, LV-1048 Riga, Latvia; jurijs.dehtjars@rtu.lv (Y.D.); elizabete.skrebele@rtu.lv (E.S.); marks.gorohovs@rtu.lv (M.G.); fricis.tenters@rtu.lv (F.T.); 2 Latvian Maritime Academy, Riga Technical University, 6B Ķīpsalas Street, LV-1048 Riga, Latvia

**Keywords:** beach sand, SEM microtextural features, XPS surface chemistry, grain roundness, sediment provenance

## Abstract

**Highlights:**

**What are the main findings?**

- Grain roundness and SEM textures differ strongly among four Latvian beach sites.
- XPS reveals distinct surface chemistry linked to abrasion, biofilms and fluvial input.
- Multi-proxy data identify unique sedimentary fingerprints for each coastal setting.

**What are the implications of the main findings?**

- Surface textures and chemistry trace sediment pathways along the Baltic coast.
- Results improve provenance interpretation in mixed coastal–fluvial environments.
- Findings support assessment of natural sands for filtration and engineering use.

**Abstract:**

Understanding the environmental pathways and surface modification of beach sand grains is essential for reconstructing coastal dynamics and assessing the suitability of natural sands for engineering applications. This study applies a multiproxy approach—integrating grain roundness classification, SEM microtextural analysis, and XPS surface chemistry—to beach sediments from four coastal sectors of Latvia: Liepaja, Ventspils, Riga, and Salacgrīva. The results reveal clear spatial differences in grain maturity, abrasion signatures, biological imprinting, and nanoscale surface composition. Liepaja is characterised by sub-rounded to rounded grains with abundant percussion pits and abrasion surfaces, indicating prolonged high-energy wave reworking. Ventspils retains angular grains with fresh conchoidal fractures, reflecting rapid sediment renewal from glacial and coastal sources. Riga exhibits weak abrasion and hydrated particulate coatings typical of low-energy brackish environments. Salacgrīva displays strong fluvial influence, including persistent diatom and algal microtextural features and elevated oxygenated carbon and metal-associated XPS signals. These findings demonstrate strong coupling between grain-surface microtextures and surface chemistry and reveal distinct sedimentary fingerprints linked to environmental setting. The multiproxy framework presented here improves understanding of Baltic coastal sediment pathways and provides a preliminary basis for future evaluation of natural sands in filtration and other environmental engineering applications.

## 1. Introduction

Littoral sediments are deposited within the coastal zone, where marine and terrestrial environments interact. This dynamic interface is governed by continuous processes of erosion, transport, and deposition [1,2,3], forming a variety of landforms and sedimentary environments. Approximately 40% of the world’s unconsolidated coastlines consist of sandy and gravelly beaches [4]. Sand is notably heterogeneous, varying in colour, grain size, and texture because of the combined influence of marine, fluvial, and aeolian processes [1,5,6,7].

Sand also represents one of the most critical industrial raw materials on Earth—second only to water in terms of global use—primarily due to its role in concrete production. However, not all sands are suitable for construction, as they must meet strict technical specifications regarding grain morphology and mineral composition [8]. Sands rich in silica (≥90%) are referred to as silica or quartz sands and are widely used in the glass, ceramics, foundry, and filtration industries [8,9,10]. Exceptionally pure deposits (99.99% SiO_2_), known as high-purity quartz sands, have strategic importance for high-technology sectors such as electronics, fibre optics, and solar energy due to their superior thermal and chemical stability [11,12,13,14].

Quartz sand represents the final product of rock weathering and a fundamental component of the sedimentary cycle. Transported mainly by water and wind, quartz grains undergo mechanical abrasion and chemical alteration, leading to rounding and compositional maturity through repeated depositional cycles [1,2,3,10,15]. Such sediments have often been investigated as archives of past environmental events, particularly through microscopic and morphometric analyses, since sand grains record specific depositional conditions and thus serve as valuable tools for paleoenvironmental reconstruction [16,17,18,19,20,21,22].

The Baltic Sea coast is particularly rich in sandy deposits, especially along its southern and eastern margins. Latvia, situated on the eastern Baltic coast, has approximately 497 km of coastline, of which 253 km lie along the Gulf of Riga [23]. Despite this, the microscopic characteristics and surface textures of coastal sands in the Baltic region remain insufficiently studied, with only limited investigations reported from Finnish and Swedish beaches [24,25].

Recognising the existing gap in current knowledge, this study investigates the sands of Latvian Baltic Sea coastal beaches located near major ports. The aim of this study is to characterise the formation and spatial variability of aeolian sediments along four distinct beach sectors of the Latvian Baltic Sea coast and the Gulf of Riga. These four sites were selected to represent the main sedimentary and hydrodynamic settings along the Latvian coastline, including the open Baltic Sea coast, the semi-enclosed Gulf of Riga, and river-influenced coastal sectors. This is achieved through an integrated analysis combining grain-size parameters, quartz grain roundness and surface microtextures (SEM), surface frosting intensity, and Fourier-transform infrared (FTIR) spectral signatures. The morphological and surface properties of quartz grains provide insights into sediment transport pathways and depositional conditions, while also providing a preliminary basis for future evaluation of these sands as potential filtration media for contaminated water (e.g., ship ballast water). Furthermore, the results contribute to defining material-selection criteria that may inform future studies aimed at improving the adsorption capacity of quartz sands in environmental remediation applications.

## 2. Materials and Methods

### 2.1. Sampling Sites of Coastal Beach Sands in Latvia

The present-day configuration of the Baltic Sea was established approximately 4500 years ago, although the formation of its coastal landscape began significantly earlier during the advance and retreat of the Scandinavian Ice Sheet. Deglaciation, shoreline fluctuations and isostatic adjustment associated with the glacial load led to the development of several transitional stages, including ice-dammed lakes (the Baltic Ice Lake and the Ancylus Lake) and marine phases (the Yoldia Sea and the Littorina Sea) [26].

Post-glacial evolution of the region has been dominated by persistent longshore currents, which transport sediments eroded from coastal outcrops and contribute to the development of extensive sandy beaches—a process that remains active today. As a result, the Latvian coastline is characterised by a relatively straight and continuous sandy shore [27].

**Figure 1:**
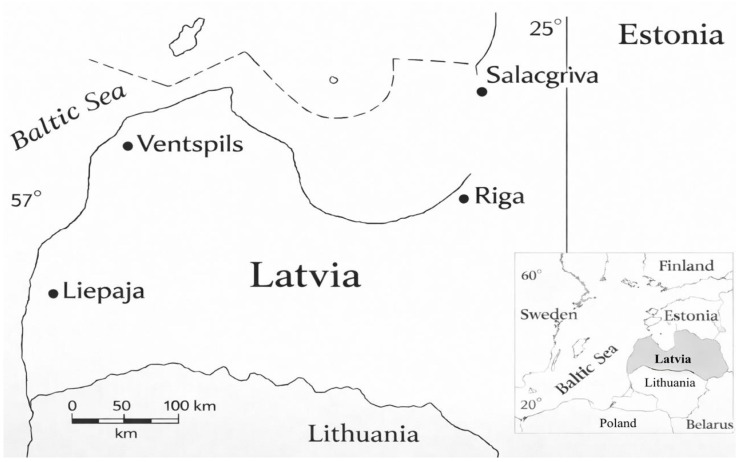
Location map of the Latvian Baltic Sea coast indicating the four main sampling sectors near the ports of Salacgrīva, Riga, Ventspils, and Liepāja; a scale bar is included.

Beach sand samples were collected from coastal sectors adjacent to the main ports of Salacgrīva (northern Gulf of Riga; 57.75° N, 24.36° E), Riga (central Gulf of Riga; 57.04° N, 24.08° E), Ventspils (north-western open Baltic Sea; 57.39° N, 21.56° E), and Liepaja (south-western open Baltic Sea; 56.52° N, 20.99° E). These four sampling locations represent the northern, eastern, central, and western sectors of the Latvian Baltic Sea coast (Figure 1).

Sampling was carried out in the coastal wave and splash zone, where aeolian and wave-driven processes are most active [28]. Sea and wind conditions were stable and comparable during the sampling period. A sampling frame was used to collect material from a 1 m^2^ area (1 m from the waterline, 1 m in width) to a depth of 30 cm. Two replicate samples were taken approximately 5 m apart within each sector. These samples represent site-level composites collected under comparable conditions and do not capture seasonal variability or broader alongshore heterogeneity. The samples were stored in sealed plastic containers, and the total mass of collected material from all sites was approximately 10 kg.

### 2.2. Beach Sand Sample Pretreatment for Grain-Size Uniformity

According to ISO 14688-1:2017 (International Organization for Standardization) on soil identification and description [29], sand is classified as fine (0.063–0.2 mm), medium (0.2–0.63 mm) and coarse (0.63–2.0 mm). This classification determined the choice of sieve mesh size used in the pre-treatment process. To remove clay, silt and other adhering fine particles, the samples were first dry-sieved using calibrated stainless-steel sieves with a mesh opening of 0.8 mm, without the aid of ultrasound [30]. The sieved material was spread evenly on trays and dried in a Nabertherm L9/B oven equipped with a proportional-integral-derivative (PID) P330 controller at 105 °C for 24 h. After drying, the samples were cooled to ambient laboratory temperature and subjected to chemical treatment to remove carbonates, iron oxides, and organic matter [31]. The grains were immersed in 15% hydrochloric acid for 15 min and then rinsed with copious deionized water to remove residual acid. Subsequently, the samples were treated with hydrogen peroxide for 20 h to oxidise organic matter, followed by repeated rinsing with deionized water until the rinse water reached near-neutral pH (pH~6.5–7.5). To minimise preparation artefacts and preserve native surface microtextures, all treatments were performed at room temperature, without ultrasonication or mechanical agitation, and with gentle decanting only. The treated material was then re-dried and dry-sieved using a stainless-steel sieve with a mesh size of 0.4 mm to obtain a uniform fine to medium grain size fraction suitable for further analysis. Special attention was paid to isolating grains with a size of <0.5 mm, as finer sand particles have a higher surface area to volume ratio, providing a greater number of active surfaces for potential interaction with dissolved contaminants such as heavy metal ions. This property is particularly important when assessing the suitability of natural quartz sand for filtration-based treatment of contaminated water, including ship ballast water.

### 2.3. Scanning Electron Microscopy (SEM)

Sand grains measuring 0.06–0.4 mm were randomly selected from the pre-treated material. An initial examination was conducted using a stereomicroscope (Nikon SMZ 800 N, Nikon Metrology, Tokyo, Japan; zoom range 1–8×; maximum magnification 80×; resolution 640 LP·mm^−1^) to verify grain integrity and assess their suitability for SEM analysis.

Randomly selected grains were placed on a transparent slide, mounted on SEM sample holders, and coated with a 15 nm gold layer to improve surface conductivity. Scanning electron microscopy was performed using a Jeol JSM-6400 (Tokyo, Japan) operated at an accelerating voltage of 15 kV. Secondary electron (SE) micrographs were obtained for each grain and processed using the freeware ImageJ v1.49 [32].

The SEM images were used for detailed qualitative analysis of grain morphology and surface microtextures. In addition, segmented grain silhouettes were used to obtain basic two-dimensional shape descriptors (projected area, perimeter, and circularity index, CI = 4πA/P^2^) for selected representative grains using a custom Python script based on the scikit-image library [33,34]. This digital image analysis served only as supplementary support for visual grain-shape assessment and quality control of segmentation, whereas the main roundness classification was based on visual evaluation from SEM images (see Section 2.5).

A microtextural feature was considered present when it occurred in more than two locations on the grain surface or covered more than 10% of the visible surface area. To obtain a representative morphological dataset, 56–72 grains were analysed.

### 2.4. Elemental and Chemical Composition Analysis

#### 2.4.1. ATR-FT-IR Spectra Acquisition

To determine the mineralogical composition of the sand samples, attenuated total reflection Fourier-transform infrared (ATR-FTIR) spectra were acquired using a Bruker Tensor II Fourier-transform infrared spectrometer (Karlsruhe, Germany) equipped with an A225-Q Platinum ATR accessory (Bruker Scientific LLC, Karlsruhe, Germany) with a diamond crystal. Data acquisition was performed using Bruker OPUS 8.0 software. Spectra were recorded in absorbance mode over the range 4000–500 cm^−1^ at a resolution of 4 cm^−1^, with 32 co-added scans per spectrum and a fresh 32-scan background acquired before each sample measurement. The interferogram size was 10,514 points with a 16k Fourier transform.

Before each background acquisition, the ATR crystal was cleaned with water followed by isopropyl alcohol and air-dried for approximately 90 s. Spectra were acquired as six independent measurements per sand type (*n* = 6) for samples originating from Salacgrīva, Liepaja, Ventspils, and Riga. Sand was poured directly onto the crystal, covered with an alumina pressure plate, and compressed using the integrated clamp to promote reproducible contact. After each measurement, the crystal was cleaned and the procedure was repeated. Automatic water compensation was enabled during acquisition, and the instrument’s default atmospheric compensation was applied.

After acquisition, the data were exported for further processing outside the OPUS environment. As the available OPUS package does not provide automatic peak (prominent local maxima of an absorption spectrum) and shoulder (less prominent spectral features occurring on the flank of a peak) detection, these functions were performed using external processing with custom Python scripts and Microsoft Excel.

Two spectral ranges were selected for feature analysis: (i) 500–1800 cm^−1^, corresponding to the dominant mineral fingerprint region for silicates and carbonates, and (ii) 2500–4000 cm^−1^, used to assess OH and CH stretching vibrations and overtone/combination bands. The 1800–2500 cm^−1^ range was considered low-confidence because the measurements showed comparatively poor signal quality, consistent with ATR-contact limitations and residual atmospheric artefacts in this region.

Spectra from the same sample type were averaged and subsequently subjected to automated feature detection. The feature-extraction pipeline consisted of six stages: (1) smoothing the spectrum to stabilise numerical operations; (2) estimating the local noise scale σ from a featureless sub-band; (3) detecting true peak maxima using a prominence criterion; (4) identifying shoulder candidates using curvature (negative second derivative) combined with an amplitude threshold; (5) de-duplicating nearby detections by merging centres within a defined tolerance; and (6) labelling each retained centre as either a “peak” or a “shoulder” for reporting and subsequent fitting.

In the first step, spectra were filtered using a Savitzky–Golay (SG) polynomial moving window (window length 31 points, polynomial order 3), which fits a low-order polynomial locally by least squares and preserves band shapes more effectively than simple moving averages for comparable noise reduction. Next, a robust estimate of the noise scale σ was computed from a sub-interval expected to contain minimal real spectral structure. In this case, the featureless 3000–3600 cm^−1^ region was selected. The estimator was based on the median absolute deviation (MAD), converted to a Gaussian-equivalent σ using the standard factor 1.4826. MAD was selected because it is substantially less sensitive than standard deviation to weak real bands or outliers during feature detection.

Peak detection was then performed, with candidate peaks defined as local maxima in the smoothed spectrum and filtered using a prominence threshold. Prominence was defined as the vertical distance from the peak to its lowest contour line determined from adjacent minima, which better reflects visually meaningful spectral bands when baselines slope or neighbouring bands overlap. The prominence threshold was defined as:

$$p_{thr}=max(6\sigma ,0.01\cdot A_{max}),$$

where *A*_max_ is the maximum absorbance in the analysed region. This combined an absolute detectability criterion with a relative amplitude threshold to prevent excessive detection of low-amplitude features in low-noise regions.

Shoulder detection was performed using second-derivative analysis, as shoulders may not form strict local maxima but produce detectable curvature changes. The second derivative was calculated using Savitzky–Golay differentiation with the same window length and polynomial order as used for smoothing. Curvature extrema were filtered using a relative derivative-prominence threshold (10% of the maximum negative second-derivative prominence in the region) and screened by requiring that the corresponding absorbance exceed 2σ. This procedure suppresses derivative-only artefacts in near-flat spectral regions.

To minimise duplicate detections of single physical bands, a minimum separation constraint of approximately 10 cm^−1^ was applied during detection. After combining peak and shoulder candidates, any centres within 8 cm^−1^ were merged into a single feature. Each retained centre was then classified as a “peak” or “shoulder” based on a relative absorbance threshold (5% of the maximum smoothed absorbance) and neighbourhood criteria within ±12 cm^−1^. All generated data were exported as Excel (.xlsx) workbooks for manual inspection and analysis.

The computational analysis was implemented in Python 3.12 using NumPy 1.26.4. [35] for array-based numerical operations, pandas for importing spectra and organising tabular data, and SciPy 1.11.4 [36] for signal-processing and optimisation routines, including Savitzky–Golay filtering and differentiation (scipy.signal.savgol_filter), prominence-based peak detection (scipy.signal.find_peaks), peak-width estimation (scipy.signal.peak_widths), and non-linear least-squares fitting for multicomponent band models (scipy.optimize.curve_fit). Output workbooks were generated using openpyxl, and file-path handling was performed using the pathlib module to ensure plat-form-independent path management.

#### 2.4.2. XPS Spectra Acquisition

X-ray photoelectron spectroscopy (XPS) measurements were performed to get additional information about the chemical composition at the surface of the sand grains. One XPS stub was prepared per sand type. Pre-treated sand was deposited onto conductive carbon tape on a molybdenum sample block, pressed gently to form a uniform layer, and excess grains were removed by light shaking. Each stub was mounted individually, and three (*n* = 3) spatially distinct positions were selected within the sand-covered area, yielding 12 measurements in total across the four sampling locations.

XPS measurements were performed using a Thermo Fisher Scientific (Waltham, MA, USA) ESCALAB Xi^+^ system with a monochromated Al Kα source. During analysis, the chamber pressure was ~10^−9^ mbar and the X-ray spot size was 900 µm. Charge neutralisation was applied using the built-in electron flood gun operated in the manufacturer’s default neutraliser mode (electron energy 40 eV, emission current 75 µA). Measurement positions were chosen to avoid exposed carbon tape, and autofocusing was performed over ±250 µm with 50 µm steps. No sputtering or etching was applied. Survey spectra were acquired from 0–1350 eV at pass energy 200 eV (1 eV step; 15 ms dwell; 4 scans). High-resolution spectra were acquired for a set of core levels (including Si 2p, O 1s, C 1s, Ca 2p, Al 2p, Mg 1s, and Fe 2p) at pass energy 30 eV (0.1 eV step; 75 ms dwell; 7 scans). Binding energy calibration (charge referencing) was performed by setting the main adventitious carbon C 1s component to 284.8 eV to correct for residual surface charging of the insulating quartz grains. Adventitious carbon was present in all samples with a broadened C 1s envelope.

### 2.5. Grain Roundness and Microtextural Classification

Sand grain roundness was evaluated visually from SEM images following the widely used Krumbein–Powers roundness scale [37], which comprises six classes: very angular, angular, sub-angular, sub-rounded, rounded, and well-rounded. Each grain was classified based on edge curvature, corner smoothness, and the degree of surface abrasion visible in secondary electron micrographs. This visual classification was applied to all analysed grains and served as the primary basis for comparative evaluation of roundness between sampling locations. To ensure internal consistency, the Krumbein–Powers classification was performed by a single trained observer; inter-observer variability was not quantified and is acknowledged as a limitation of this exploratory study. Surface microtextures were classified into four major genetic categories based on established criteria reported in previous studies [38,39,40,41]. A microtextural feature was considered present when it occurred in more than two locations on the grain surface or covered more than 10% of the visible surface area. The following interpretation scheme summarizes typical associations between visual roundness classes and dominant surface microtextures observed in beach sand grains and used to infer sediment transport processes and depositional environments. The interpretation scheme summarizing the associations between visual roundness classes, diagnostic microtextural indicators, and inferred transport environments is presented in Table 1.

The combined visual roundness classes and microtextural features were used to infer sediment transport history, abrasion intensity, and the degree of aeolian reworking among the four coastal sectors, as well as to assess the potential suitability of grains for use in filtration systems.

## 3. Results

### 3.1. Sand Grain Roundness Analysis

Visual classification of 272 sand grains based on the Krumbein–Powers roundness scale shows that sub-angular and sub-rounded grains dominate at all four sampling locations (Liepaja, Riga, Salacgrīva and Ventspils). Very angular grains are rare, while rounded and well-rounded grains occur only as minor components. This overall distribution indicates moderate mechanical abrasion and repeated sediment reworking rather than prolonged aeolian or persistent high-energy coastal transport.

In the Liepaja samples, most grains fall within the sub-angular to sub-rounded classes, with occasional rounded and well-rounded grains. This suggests mixed sediment sources and local reworking, likely influenced by nearshore processes, shell fragments, and lithologically distinct particles contributing locally rounded material.

Riga samples are characterized mainly by angular to sub-angular grains, with fewer sub-rounded particles. This pattern is typical of river-dominated sediment supply, where mechanical abrasion is limited and sediment residence time is relatively short.

Ventspils samples display a broader range of roundness classes, including a higher proportion of sub-rounded and rounded grains compared to Riga. This indicates additional local reworking, likely related to coastal wave action, shoreline processes, or port-related sediment disturbance.

In the Salacgrīva samples, sub-angular and sub-rounded grains dominate, but rounded grains are also present. This combination reflects mixed transport histories, where fluvial input is modified by subsequent coastal reworking.

Overall, two broadly similar sedimentary tendencies can be distinguished. Liepaja and Salacgrīva show stronger nearshore modification of fluvial sediments, whereas Riga retains clearer fluvial signatures, and Ventspils exhibits mixed characteristics reflecting both fluvial input and additional coastal reworking. These differences indicate that local hydrodynamic conditions and shoreline morphology play a major role in controlling final grain-shape characteristics along the coast. The comparisons are based on observed frequency patterns within the analysed grain set and are therefore interpreted as qualitative trends in this exploratory study.

### 3.2. SEM Microtextural Analysis of Sand Grains

Beach sand deposits along the Latvian coast are derived primarily from glacial and postglacial sediments associated with the Baltic Ice Lake and moraine formations. However, the surface microtextural features observed in the SEM images indicate that most grains have undergone substantial modification in the modern coastal environment. The dominance of abrasion-related features suggests repeated reworking by wave action and nearshore transport processes, which overprint many of the original glacial signatures. As a result, the preserved microtextures mainly reflect recent coastal processes rather than primary depositional environments. The occurrence and relative abundance of the identified microtextural features across the four sampling locations and their dominant process interpretations are summarized in Table 2.

**Figure 2:**
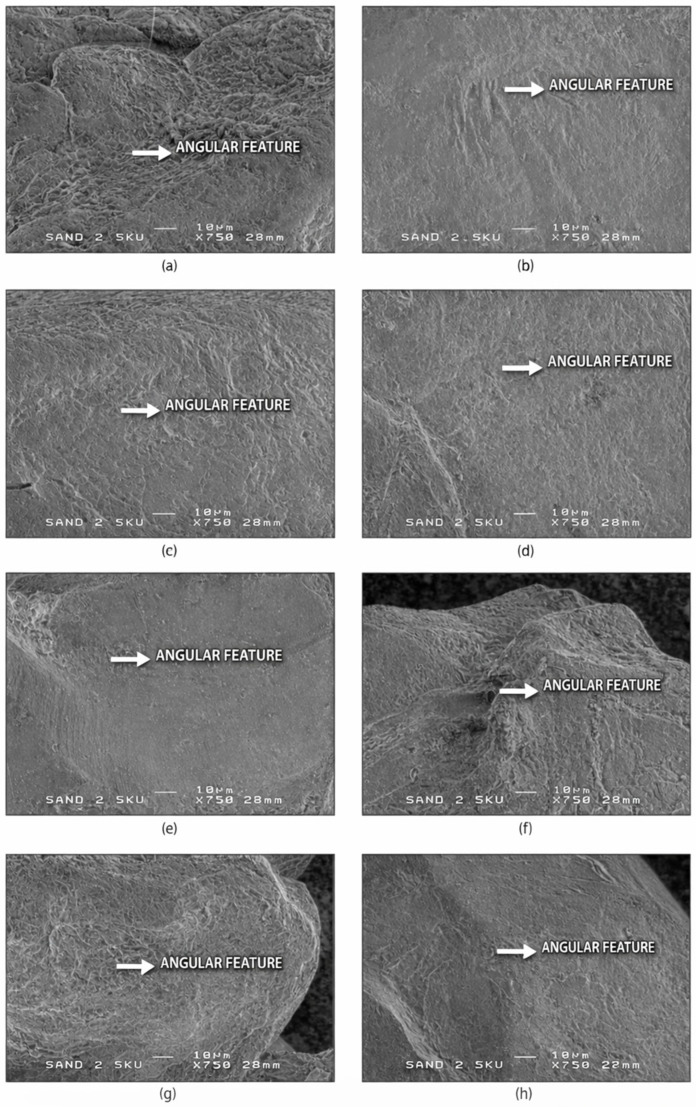
SEM-1—Angular microtextural features in quartz sand grains. Representative SEM images (**a**–**h**) of angular quartz sand grains showing sharp, high-relief edges, flat detrital fracture surfaces, preserved conchoidal fracture steps, and an absence of mechanical rounding. These microtextural features indicate minimal transport modification and preservation of fresh fracture surfaces, consistent with early-stage mechanical fragmentation prior to significant coastal reworking. White arrows mark diagnostic angular features. Scale bar = 10 µm.

SEM analysis reveals characteristic surface microtextures that are sensitive to mechanical abrasion and chemical modification. These features serve as reliable proxies for reconstructing sedimentary histories, as they persist through multiple reworking cycles. The six SEM groups identified in this study form a progression from mechanically pristine to strongly abraded grains and are described below, starting with the least mature and most environmentally diagnostic class (SEM-1). Representative SEM images of angular quartz grains and their diagnostic microtextural features (SEM-1) are shown in Figure 2.

**Figure 3:**
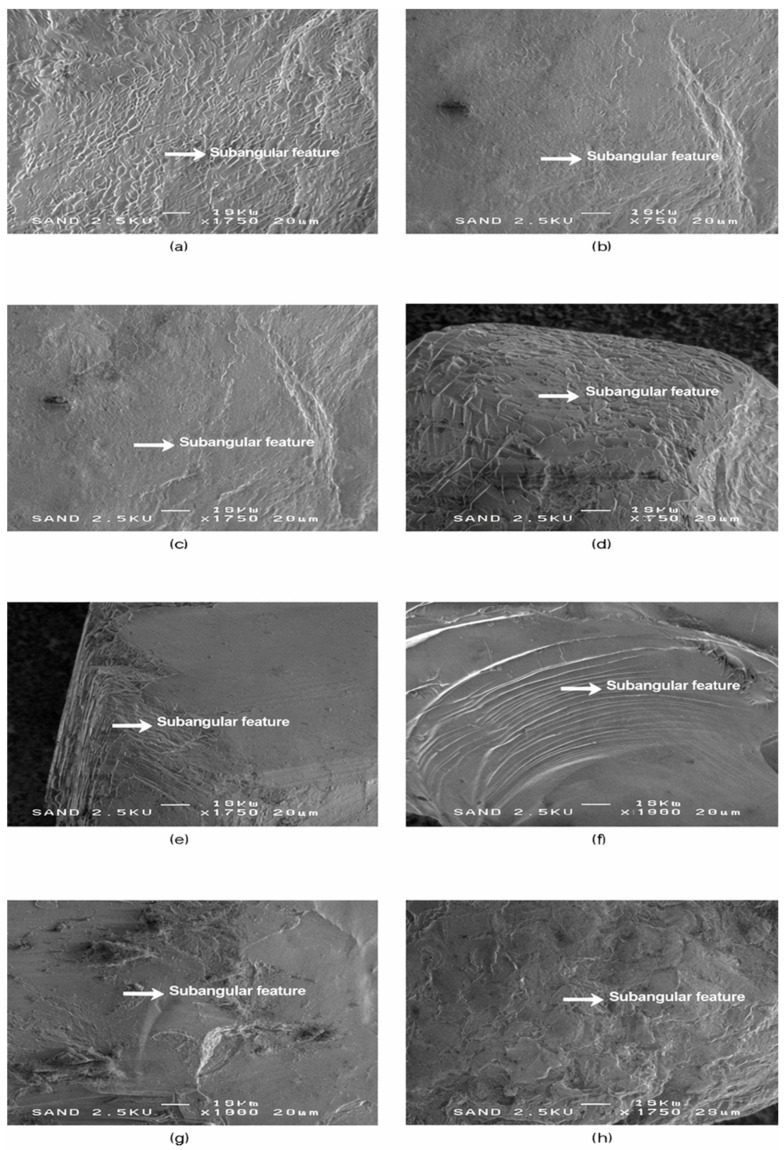
SEM-2—Sub-angular microtextural features in quartz sand grains. Representative SEM images (**a**–**h**) of sub-angular quartz sand grains showing residual fracture facets combined with incipient edge smoothing and the development of micro-steps, indicating the onset of mechanical rounding. This group represents moderately weathered grains and includes samples from all four coastal sectors. Most sub-angular grains are from Riga (**a**–**c**) and Salacgrīva (**d**,**e**), with additional examples from Ventspils (**f**) and Liepaja (**g**,**h**). These microtextural features reflect partial modification during transport and early stages of coastal reworking. White arrows mark diagnostic sub-angular features. Scale bar = 20 µm.

Following the angular grains described in SEM-1, the second microtextural group (SEM-2) comprises sand grains showing incipient abrasion and partial edge modification, consistent with short to intermediate transport. Representative images and diagnostic microtextural features of this group are shown in Figure 3.

**Figure 4:**
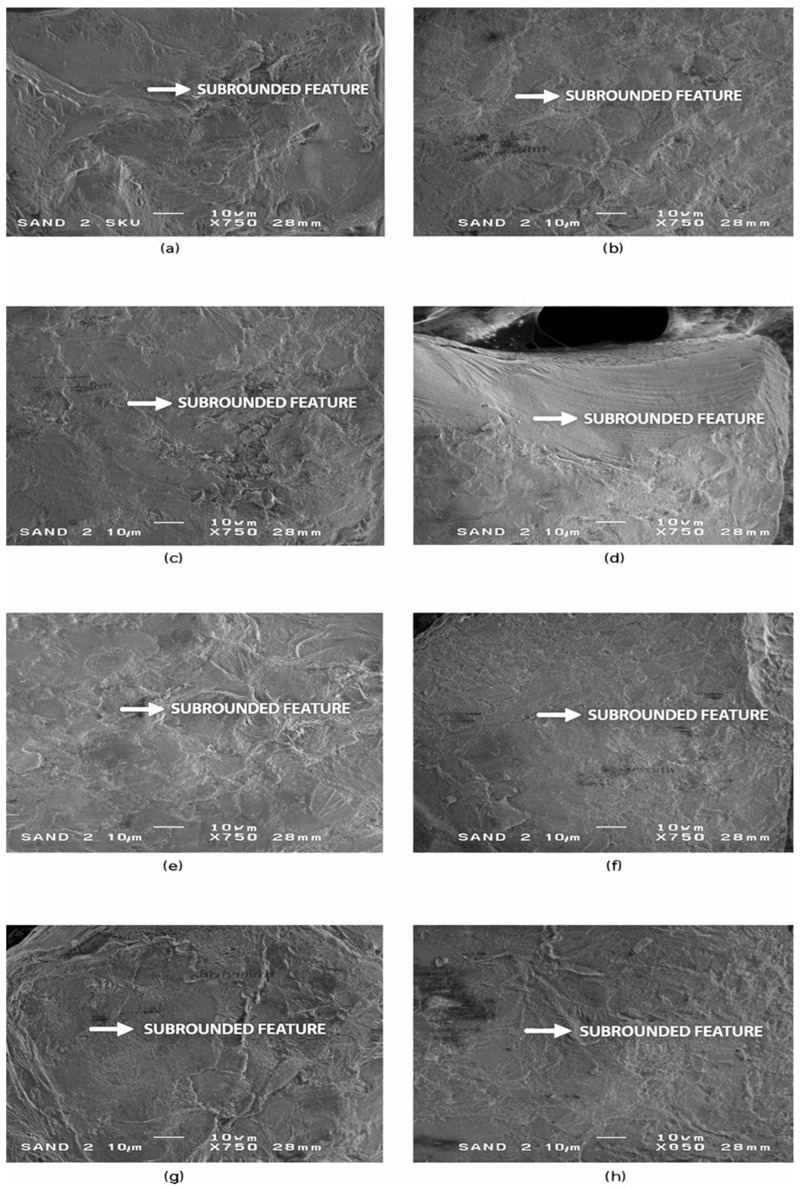
SEM-3—Sub-rounded microtextural features in quartz sand grains. Representative SEM images (**a**–**h**) of sub-rounded quartz sand grains showing moderate abrasion, well-developed edge smoothing, shallow mechanical impact pits, and patchy surface polish. Panels (**a**–**c**) illustrate grains from Liepaja, panels (**d**,**e**) from Ventspils, and panels (**f**–**h**) from Salacgrīva. These microtextural features indicate prolonged littoral reworking, consistent with repeated wave-driven grain collisions and sediment recycling in higher-energy coastal environments. White arrows mark diagnostic sub-rounded features, including shallow percussion pits, polished facets, and uniformly abraded edges. Scale bar = 10 µm.

Moving beyond the sub-angular grains, the third microtextural group (SEM-3) consists of sub-rounded quartz grains showing more advanced abrasion and mechanical smoothing. In contrast to SEM-1 and SEM-2, this group does not include grains from Riga, indicating pronounced spatial variability in sediment reworking and a stronger influence of coastal transport processes at the remaining sites. Representative SEM images and diagnostic microtextural features of this group are shown in Figure 4.

**Figure 5:**
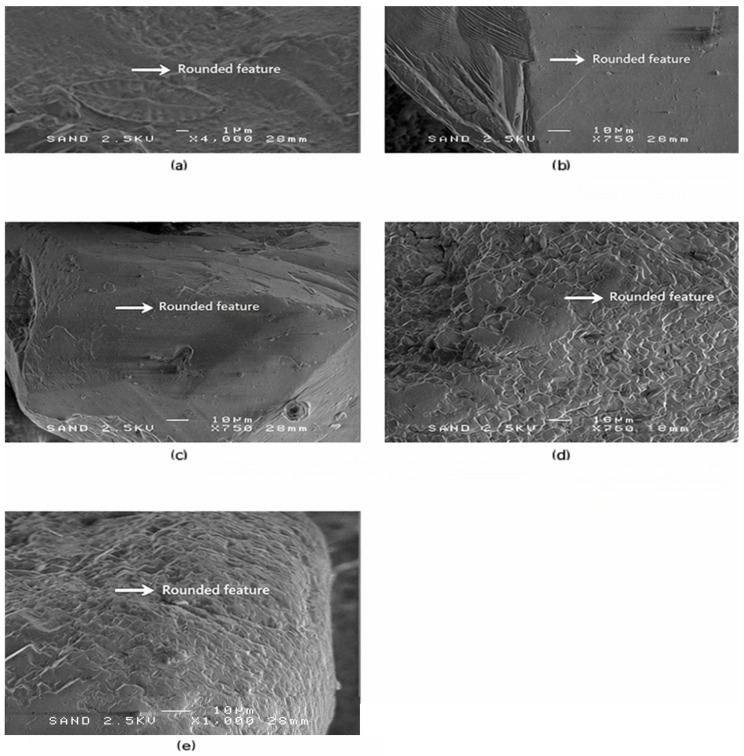
SEM-4—Rounded microtextural features in quartz sand grains. Representative SEM images (**a**–**e**) of well-rounded and highly abraded quartz sand grains reflecting prolonged sediment reworking in energetic coastal environments. Panels (**a**–**d**) illustrate grains from Liepaja, and panel (**e**) shows a grain from Ventspils. The grains exhibit extensive edge smoothing, low-relief polished surfaces, and strongly curved fracture boundaries, which are characteristic of longshore transport and repeated surf-zone abrasion. White arrows highlight diagnostic rounded microtextural features, including polished facets and uniformly abraded edges. Scale bars = 1 µm in (**a**) and 10 µm in (**b**–**e**).

The fourth microtextural group (SEM-4) represents a further progression toward rounded grain morphologies typical of mature, repeatedly reworked coastal sediments. This group contains only five grains, originating from Liepaja and Ventspils. Their surface characteristics show extensive smoothing and low-relief, well-polished surfaces, consistent with longshore transport and multiple sediment recycling cycles. Representative SEM images and diagnostic microtextural features of SEM-4 are shown in Figure 5.

**Figure 6:**
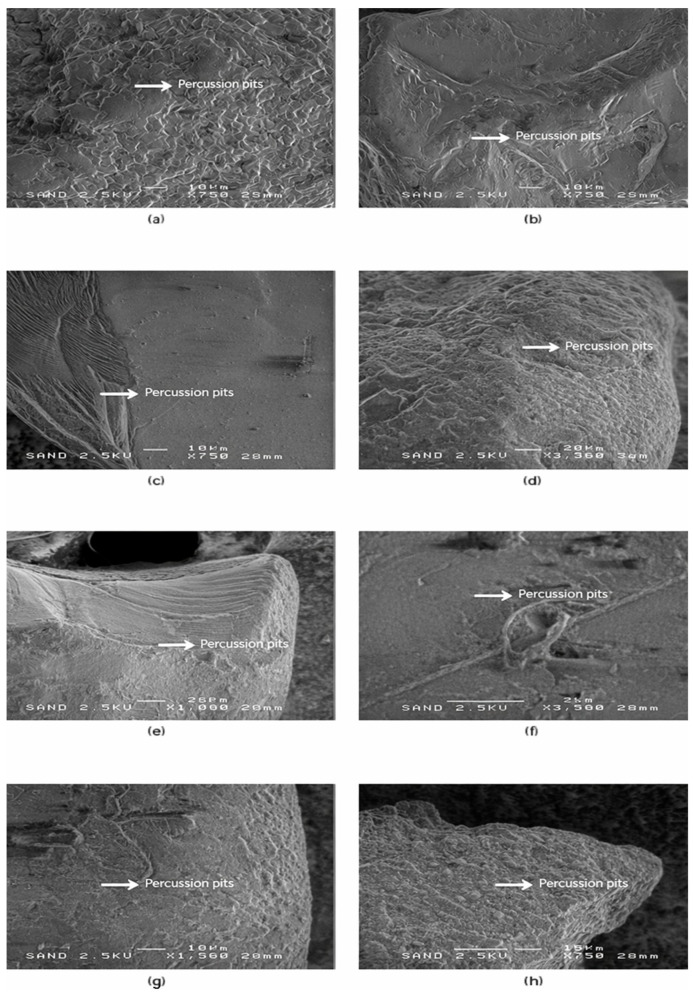
SEM-5—Percussion pits and impact-related microtextural features in quartz sand grains. Representative SEM images (**a**–**h**) of quartz sand grains showing well-developed mechanical impact structures, including circular to sub-elliptical percussion pits, pit clusters, and overlapping impact scars. Panels (**a**–**c**) show grains from Liepaja, panels (**d**,**e**) from Ventspils, and panels (**f**–**h**) from Salacgrīva. The abundance and density of impact pits indicate intense high-energy grain–grain collisions, consistent with repeated mobilisation in surf-zone and nearshore settings. White arrows indicate representative impact scars and percussion pit morphologies. Scale bars = 10 µm in (**a**–**c**,**g**,**h**) and 20 µm in (**d**–**f**).

Progressing beyond the rounded grains of SEM-4, the fifth microtextural group (SEM-5) includes grains that display distinct mechanical impact features, such as circular and elongated percussion pits and overlapping impact scars. Unlike the previous groups, SEM-5 does not include grains from Riga, reflecting spatial differences in sediment reworking intensity. These features are indicative of high-energy grain–grain collisions, typical of surf-zone turbulence and sediment reworking under strong wave forcing. Representative SEM images and diagnostic impact-related microtextural features of this group are shown in Figure 6.

**Figure 7:**
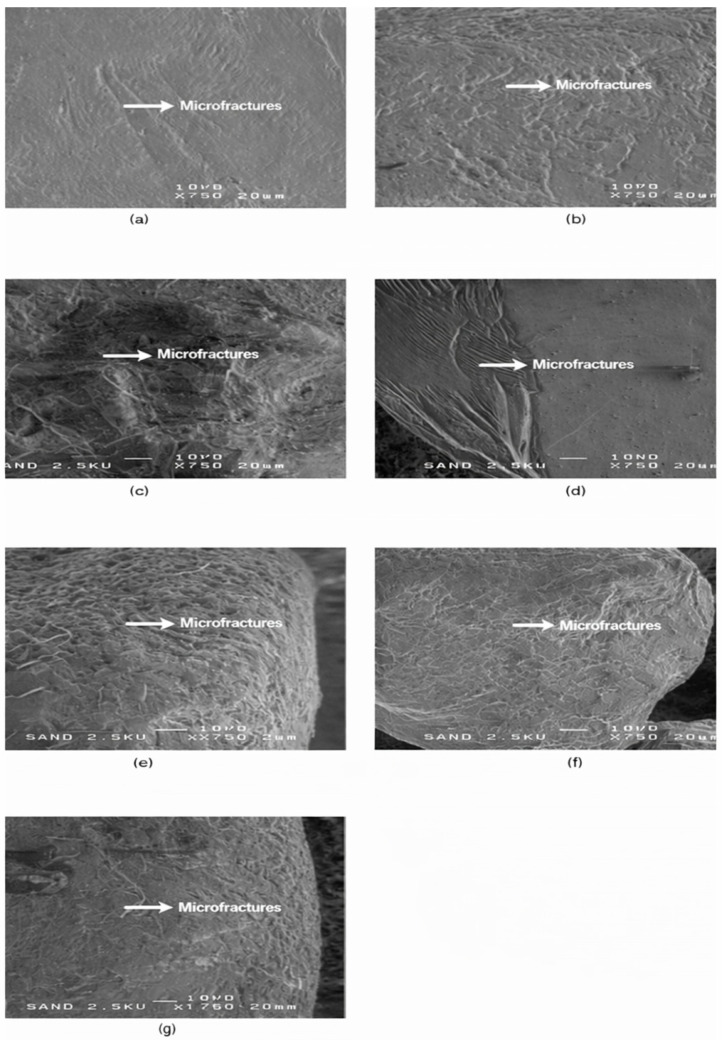
SEM-6—Conchoidal microcracks and microfractures in quartz sand grains. Representative SEM images (**a**–**g**) of quartz sand grains exhibiting brittle mechanical damage, expressed as progressive fracture propagation, microfractures, jagged edges, and conchoidal fracture facets. Panels (**a**,**b**) show grains from Riga, panels (**c**,**d**) from Liepaja, panel (**e**) from Ventspils, and panels (**f**,**g**) from Salacgrīva. These microtextural features record episodic high-stress grain–grain collisions and damage propagation along pre-existing microstructural weaknesses. White arrows indicate diagnostic fracture and microfracture zones used to distinguish brittle impact features from abrasion-dominated surfaces. Scale bar = 20 µm.

The sixth microtextural group (SEM-6) includes grains from all four sampling locations and is characterized by microfractures, conchoidal breakage and microchipping, indicating brittle mechanical failure during transport. The small number of grains suggests episodic high-energy conditions, such as storm-driven impacts or mobilisation over hard substrates. Representative SEM images and diagnostic brittle-impact microtextural features of this group are shown in Figure 7.

Together, the SEM observations and visual roundness classification demonstrate clear spatial differences in abrasion intensity and sediment maturity across the four coastal sectors. To further investigate the chemical composition and surface functional groups of the quartz grains, complementary spectroscopic analyses were performed. The following section integrates X-ray photoelectron spectroscopy (XPS) and Fourier-transform infrared spectroscopy (FTIR) to characterise the mineralogical and surface-chemical signatures associated with the observed microtextural features.

### 3.3. Spectroscopic Characterisation of Quartz Grains (XPS and FTIR)

#### 3.3.1. Fourier-Transform Infrared Spectroscopy

**Figure 8:**
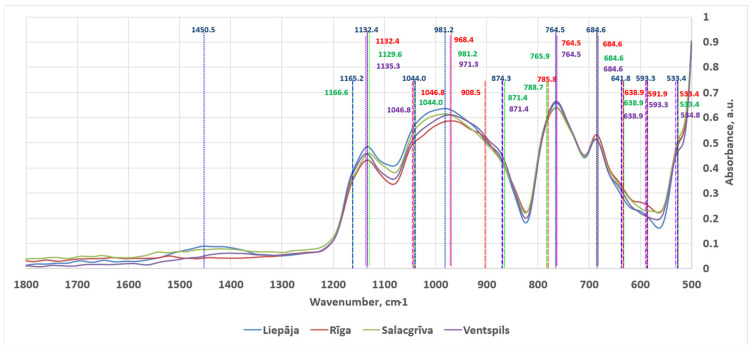
ATR-FT-IR spectra in the 1800–500 cm^−1^ region acquired for sands from Liepaja, Riga, Salacgrīva and Ventspils. The long, small-dashed lines mark peaks, while the short, large-dashed lines represent shoulders.

**Figure 9:**
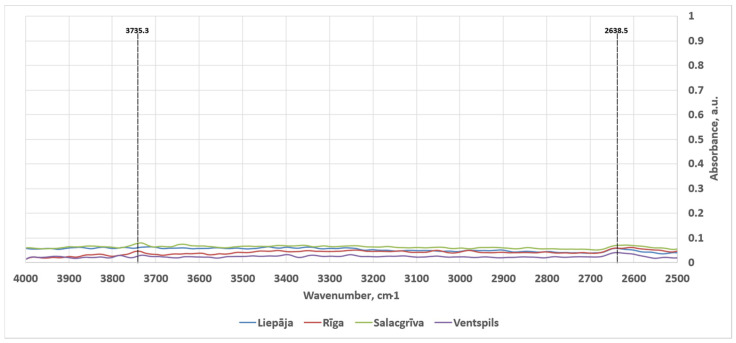
Averaged, normalized ATR-FT-IR spectra in the 4000–2500 cm^−1^ region acquired for sands from Liepaja, Riga, Salacgrīva and Ventspils. The long, small-dashed lines mark peaks.

Across all four sampling locations (Liepaja, Riga, Salacgrīva, Ventspils), the spectra were dominated by the broad framework-silicate envelope spanning approximately 1200–800 cm^−1^ as can be seen in Figure 8. The 2500–4000 cm^−1^ spectral region was mostly empty save for two small peaks. The averaged, normalized spectra in this high-wavenumber region are shown in Figure 9. The spectra are consistent with quartz–feldspar rich sands where multiple Si–O stretching contributions overlap and individual minerals contribute sub-features rather than isolated, uniquely diagnostic peaks in mixtures. The detected features in the interpreted regions are summarized below using the final feature list provided in Table 3 (including the three barely visible shoulder intervals marked with *). Band assignments are stated as the most compositionally plausible interpretations for beach sand mixtures rather than single-mineral identifications.

#### 3.3.2. X-Ray Photoelectron Spectroscopy

Survey XPS spectra were dominated by O 1s and Si 2p/Si 2s signals, consistent with oxidized silicon environments on the outermost surface. Adventitious carbon was present in all samples as a prominent C 1s envelope. Lower intensity features attributable to common inorganic constituents and salts were also observed (Al 2p, Na 1s, Mg 1s, Ca 2p, Fe 2p, K 2p, Cl 2p, and N 1s), with relative intensities varying between sampling locations.

**Figure 10:**
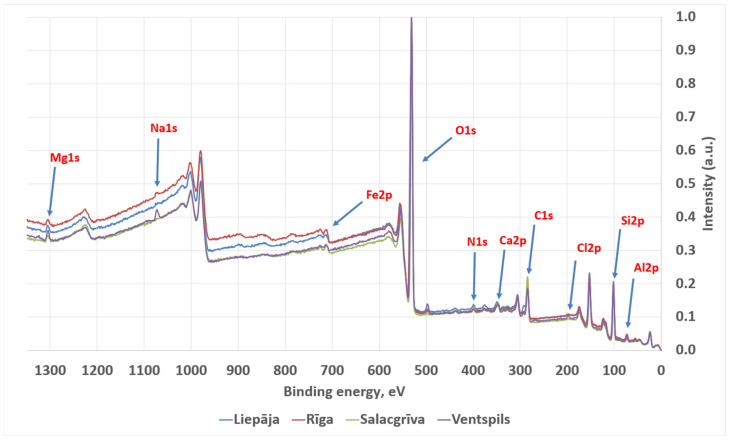
Averaged, normalized XPS spectra for sand samples from Liepaja, Riga, Salacgrīva, and Ventspils.

**Figure 11:**
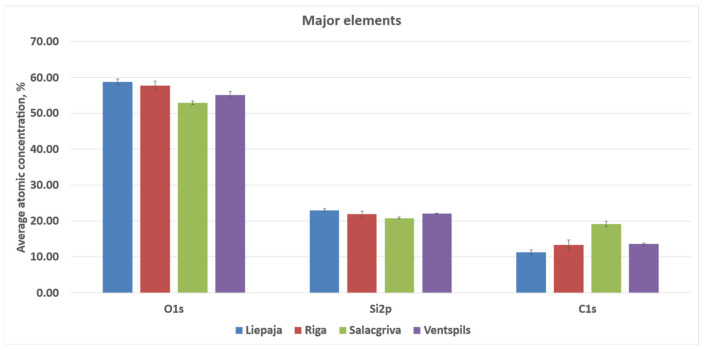
Elements with a higher atomic % contribution to the elemental composition of the studied sand samples from Liepaja, Riga, Salacgrīva, and Ventspils.

The calculated atomic concentrations from the survey spectra are as follows: O 1s = 51.9–59.4 at%, Si 2p = 23.2–27.2 at%, C 1s = 11.1–20.6 at%, Al 2p ≈ 3.3–4.3 at%, Na 1s ≈ 0.6–1.2 at%, Ca 2p ≈ 0.15–0.31 at%, Mg 1s ≈ 0.05–0.17 at%, Fe 2p ≈ 0.09–0.31 at%, Cl 2p ≈ 0.31–0.56 at%, and N 1s ≈ 0.11–0.26 at%. These atomic concentrations are treated as semi-quantitative indicators for relative surface comparison; no high-resolution chemical-state deconvolution (e.g., C 1s peak fitting) was performed. Carbon was highest for Riga (20.6 ± 1.0 at%) and lowest for Salacgrīva (11.1 ± 0.5 at%). Elemental ratios derived from the quantified survey compositions showed O/Si ≈ 2.49–2.63 and C/Si ≈ 0.47–0.81 (means), with total non-Si cations relative to Si (Σ(Al + Ca + Mg + Fe + Na + K)/Si) ≈ 0.16–0.24 across the four sands. Average concentrations for sand samples from all four regions are presented in Figure 10 and Figure 11.

**Figure 12:**
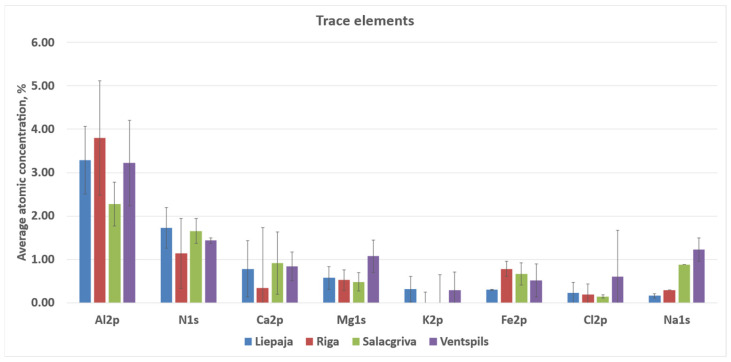
Elements with a lower atomic % contribution to the elemental composition of the studied sand samples from Liepāja, Rīga, Salacgrīva, and Ventspils.

**Figure 13:**
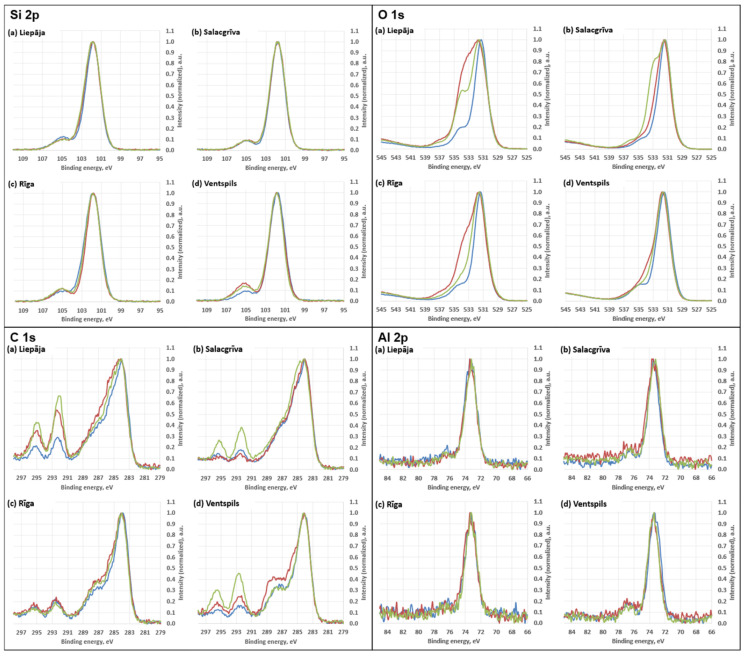
High-resolution spectra for silicon (**Si 2p**), oxygen (**O 1s**), carbon (**C 1s**), and aluminium (**Al 2p**). Different coloured curves represent spectra acquired at different measurement positions on the same sample.

**Figure 14:**
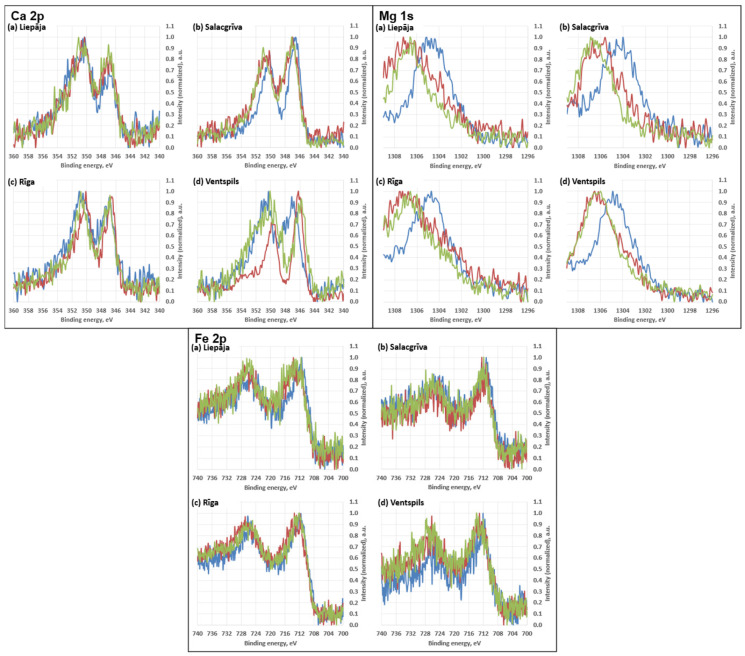
High-resolution spectra for calcium (**Ca 2p**), magnesium (**Mg 1s**), and iron (**Fe 2p**). Different coloured curves represent spectra acquired at different measurement positions on the same sample.

The high-resolution regions (Figure 12 and Figure 13) showed envelopes consistent with mixed silicate surfaces and possible carbonaceous contributions. High-resolution spectra for Ca 2p, Mg 1s, and Fe 2p are shown in Figure 14. Features in the higher-binding-energy portion of C 1s (near the carbonate region) and slight variations in the O 1s envelope may be consistent with minor carbonate-type surface contributions; however, no chemical-state deconvolution was performed, and these observations are interpreted qualitatively.

#### 3.3.3. Spectral Data Analysis

The FT-IR signatures collectively support a framework-silicate-dominated mineralogy in all four regions, with quartz and feldspar as the most plausible principal phases. The band observed at 684.6 cm^−1^ lies close to the widely used quartz diagnostic region at ~690–700 cm^−1^ [42,44,45,46]. The shift in the apparent maximum to 684.6 cm^−1^ is consistent with the expected behaviour of quartz bands in mixtures and under ATR contact conditions, where overlap with other silicate modes and particle–crystal contact effects can shift apparent maxima and alter relative intensities [45,46]. Quartz mixture diagnostics are commonly discussed using the ~695 cm^−1^ feature together with the ~798 and ~779 cm^−1^ bands.

The persistent low-wavenumber shoulders at 533–534, 591–594, and 638–642 cm^−1^ are most consistent with feldspar (and other framework-silicate) lattice/deformation contributions rather than quartz-unique markers [42]. Comparative K-feldspar spectra (microcline/orthoclase/sanidine) report band sets that include features in the ~640–646 cm^−1^ and ~584–604 cm^−1^ regions, with additional feldspar bands appearing across the 800–1200 cm^−1^ Si–O stretch domain [43]. The composite envelope between ~1200 and 800 cm^−1^ (including the 971–983 peak and the 1044–1047 and 1090–1110 shoulders) is therefore interpreted as the expected superposition of quartz and feldspar Si–O stretching sub-structure rather than a single mineral-diagnostic band [43].

The spectra have traces of carbonate-based compounds. Two FT-IR features provide the clearest carbonate evidence: the ν_2_ out-of-plane bend at 871–875 cm^−1^ (present in Liepaja, Salacgrīva, and Ventspils) and the ν_3_ asymmetric stretch band at 1450.5 cm^−1^ (present in Liepaja and visually discernible in Salacgrīva). Carbonate minerals such as calcite and dolomite are well documented to exhibit strong ν_2_ features near ~875 cm^−1^ and ν_3_ structure in the ~1400–1500 cm^−1^ region, with additional carbonate bands near ~712–713 cm^−1^ (ν_4_) that can contribute to the 725–750 cm^−1^ overlap interval [48,49]. However, the 725–750 cm^−1^ shoulder is best treated as an overlap zone rather than a single-phase identifier. In mineral mixtures, this interval can contain contributions from carbonates (ν_4_ region, e.g., calcite near ~712 cm^−1^ and dolomite/aragonite nearer ~728–730 cm^−1^) [48,49] and from feldspar-related modes discussed near ~726 cm^−1^ in K-feldspar band sets [43,44,47]. On this basis, the FT-IR spectra indicate that carbonate is most consistently supported in Liepaja and Salacgrīva (ν_2_ present and ν_3_ clearly expressed) and is also present at trace levels in Ventspils (ν_2_ present), with Riga showing little to no carbonate presence.

The weak feature at 2638.5 cm^−1^ was present in all samples but is not, by itself, a standard diagnostic maximum for the most common sand minerals. Carbonates can show overtone/combination structure in the 2500–2600 cm^−1^ region (for example near ~2513–2525 cm^−1^) [42], but the observed position at 2638.5 cm^−1^ falls outside the typical maxima emphasized for calcite/dolomite in mixture-focused references and is therefore best treated as non-diagnostic without corroboration from stronger carbonate bands.

The qualitative XPS survey spectra are consistent with this interpretation, as Ca 2p (and weaker Mg 1s, where present) signals occur alongside the dominant silicate peaks, compatible with minor carbonate and/or carbonate-bearing grain surfaces. In the absence of quantitative analysis and detailed chemical-state separation of the C 1s, O 1s, and Ca 2p regions, XPS is used here only as corroborative evidence that carbonate-related elements are present on the analysed grain surfaces, rather than as a phase-specific discriminator.

The FT-IR shoulders at ~910–930 cm^−1^ across all samples, together with the distinct 908.5 cm^−1^ shoulder unique to Riga, are compatible with aluminosilicate/clay contributions in mixed mineral spectra [44]. Published mineral-mixture and clay-mineral FT-IR analyses frequently discuss features in the ~915–925 cm^−1^ region as arising from Al–OH deformation and/or coupled silicate vibrations in clay and mica families (with the exact position and prominence varying with mineralogy and mixture context) [44]. The presence of Al 2p in the XPS survey spectra across samples provides independent support for aluminosilicate material at the outermost surface, consistent with feldspars and/or clay/mica phases in the sand matrix.

The weak OH feature at 3735.3 cm^−1^ (Riga and Salacgrīva) is consistent with isolated or weakly hydrogen-bonded silanol (Si–OH) groups reported near ~3747 cm^−1^ on silica/silicate surfaces, with the intensity of this band known to depend on surface hydration state and pretreatment [50,51,52]. Its absence in Liepaja and Ventspils suggests a lower abundance of isolated silanol on the measured surfaces under the acquisition conditions, potentially reflecting differences in surface area, surface cleanliness, salt/organic coverage, or the distribution of freshly fractured versus weathered grain surfaces. The qualitative XPS observation of chemically broadened O 1s envelopes across samples is compatible with mixed oxygen environments (siloxane and hydroxylated oxygen on silicates, with possible carbonate oxygen where Ca is present).

Taken together, ATR-FT-IR and survey-level XPS converge on a consistent compositional picture: all four beach sands are dominated by framework silicates (quartz + feldspars), with variable but generally minor contributions from carbonates and aluminosilicate/clay-like components. XPS adds that these components are expressed at the immediate grain surface, where adventitious carbon and salt-type species (Na, Cl) are also present and may mask or attenuate minor mineral signatures. Interpretation is constrained by method-specific limitations: ATR-FT-IR intensities in granular media depend strongly on grain–crystal contact and particle size [53,54], and XPS in the present workflow was not used for chemical-state fitting or quantitative compositional analysis.

## 4. Discussion

This study demonstrates that quartz grain roundness, SEM microtextural features, and XPS surface chemistry together provide strong constraints on sediment provenance, mechanical history, and environmental modification along the Latvian Baltic Sea coast. Although the bulk mineralogy of all samples is dominated by quartz, the surfaces of individual grains retain highly diagnostic information on abrasion, biological colonisation, fluvial influence, and chemical weathering. These multiproxy signatures vary systematically among the four study sites—Liepaja, Ventspils, Riga, and Salacgrīva—reflecting contrasting hydrodynamic regimes, sediment budgets, and geomorphological settings.

### 4.1. Mechanical Abrasion and Sediment Maturity Along the Open Baltic Coast

A key finding of this study is the pronounced contrast between the two open-coast environments, Liepaja and Ventspils. Despite their similar exposure to high-energy wave fields, grains from Liepaja exhibit consistently higher roundness and abrasion than those from Ventspils in the analysed samples. SEM microtextural features from Liepaja include well-developed percussion pits, crescentic fractures, smoothed edges, and low-relief abrasion surfaces—features consistent with longshore drift reworking, oscillatory wave polishing, and multiple transport cycles. These textures closely correspond to classic high-energy coastal signatures described by [40], who demonstrated that wave agitation promotes uniform rounding and pit development. Likewise, ref. [38] identified similar features in mature beach sediments worldwide, emphasising the cumulative effects of repeated mechanical impacts.

In contrast, Ventspils grains commonly exhibit sharp angular outlines with conchoidal fractures, stepped edges, and brittle microchipping. These are diagnostic features of freshly fragmented glacial sediments, characteristic of paraglacial systems where sediment supply exceeds abrasion time. Such grains were described by [39] as “mechanically immature”, a characterisation consistent with the Ventspils sediments. Notably, this pattern persists despite strong wave exposure, indicating that abrasion efficiency depends not only on hydrodynamic energy but also on grain residence time. Similar observations were reported by [41], who showed that beaches receiving continuous sediment input may retain angular grains even under high-energy wave conditions. Our results support this model, indicating that Ventspils functions as a high-energy system with low sediment maturity due to continuous sediment supply.

### 4.2. Biological Imprinting and Organic Surface Coatings in Transitional Coastal Systems

Biological microtextural features—including diatom frustule impressions, algal residues, microbial pits, and extracellular polymeric substance (EPS) films—occur primarily in Liepaja and Salacgrīva. These microstructures are significant because they indicate not only biological activity but also the persistence of surface coatings. SEM images show intact diatom impressions and EPS-like matrices, suggesting that quartz grains remain in nearshore or shallow-water environments for sufficient periods to allow biofilm colonisation.

XPS data confirm these observations chemically: both Liepaja and Salacgrīva exhibit elevated proportions of oxygenated carbon species (C–O, C=O, O–C=O) and broad OH-related O 1s components, consistent with organic polymeric coatings and hydrated biofilms. Such signatures are widely recognised in studies of biologically modified sediments [55,56,57].

A key distinction between the two sites is that Liepaja shows episodic biofilm retention, consistent with periodic mechanical abrasion removing surface coatings, whereas Salacgrīva displays more persistent biological signatures. This contrast reflects site-specific hydrodynamic and water-quality conditions. The Liepaja sector is exposed to higher wave energy and more frequent storm-driven resuspension, which increases near-bed shear stress and promotes repeated removal of weakly bound surface films, as documented by regional coastal-process monitoring and erosion/accumulation studies for the Gulf of Riga and adjacent open-coast sectors [58,59,60,61]. In contrast, the Salacgrīva coast is strongly influenced by river discharge and associated elevated loads of suspended and organic matter, including diatoms and fine colloids, which favour continuous supply, adhesion, and retention of biogenic and organic coatings on quartz grain surfaces. Similar behaviour has been reported from river-influenced Baltic coastal systems, where lower-energy conditions combined with higher turbidity promote persistent biological and organic surface films [59].

### 4.3. Hydrated Particulate Coatings in Low-Energy Brackish Basins

Riga presents a markedly different surface signature from the other three sites. Grain roundness is low, abrasion features are poorly developed, and SEM reveals no structured biogenic imprinting. Instead, the dominant surface feature is a diffuse hydrated particulate coating. XPS spectra display broad OH components and weakly oxidised carbon, consistent with thin clay-rich or fine suspended sediment films rather than organised EPS coatings. Similar spectral signatures have been reported for mineral grains coated by hydrated silicate layers [58].

These observations are consistent with sedimentation studies in the Gulf of Riga [60,61], which documented limited wave recycling, high retention of suspended sediments, and reduced mechanical abrasion. The semi-enclosed hydrography and brackish-water conditions promote the settling of fine particles onto quartz grain surfaces, explaining the observed chemical signatures.

### 4.4. Insights from Baltic Regional Studies: Provenance, Sediment Pathways, and Environmental Controls

The differentiation among the four Latvian coastal sectors fits well within broader sediment evolution frameworks for the Baltic Sea. A regional synthesis by [62] identified three primary controls on coastal sediment characteristics: (i) sediment supply, (ii) wave-regime intensity, and (iii) geomorphological confinement. These controls correspond closely to the patterns observed in this study:

**Liepaja**—high wave energy and low sediment input → mature, rounded grains

**Ventspils**—high wave energy and high sediment input → angular, freshly fractured grains

**Riga**—low wave energy and strong confinement → hydrated particulate coatings

**Salacgrīva**—moderate energy and riverine supply → persistent biological coatings

Multimethod Baltic Sea studies [59] similarly demonstrate that combined microtextural and geochemical indicators can differentiate sediment sources and transport pathways, producing distinct mineral-surface fingerprints. This multiproxy behaviour is directly reflected in the present study. Furthermore, persistent organic coatings observed in Salacgrīva correspond to patterns reported from river-dominated Lithuanian beach systems [61], while the angular and mechanically immature grain signatures at Ventspils are consistent with regional geological maps and coastal-process syntheses documenting active glacial and paraglacial sediment supply along the Latvian Baltic Sea coast [23].

### 4.5. Microtextural–Surface Chemistry Coupling as an Environmental Proxy

A major strength of this study is the consistency between physical and chemical grain-surface indicators. The coupling between SEM microtextural features and XPS chemistry provides a robust diagnostic framework:

- Rounded, abrasion-smoothed grains (Liepaja) → moderate oxidised carbon with OH components.
- Angular, freshly fractured grains (Ventspils) → comparatively sharper Si 2p envelopes and lower relative carbon content.
- Diffuse hydrated coatings (Riga) → broad OH components and weak C–O signals.
- Biogenic coatings (Salacgrīva) → strong C–O and C=O components and elevated trace metals.

These results demonstrate that nanoscale surface chemistry tracks the same environmental processes identified from SEM observations, supporting the integration of XPS into coastal sediment provenance studies, which remain relatively rare in the Baltic Sea region.

### 4.6. Implications for Sediment Provenance, Coastal Evolution, and Filtration Potential

**Sediment provenance.**

The combination of angularity, fracture patterns, and chemically clean quartz surfaces in Ventspils indicates active glacial or paraglacial sediment supply. In contrast, the mixed biological–mechanical signatures in Salacgrīva reflect combined fluvial and marine influences.

**Coastal evolution.**

Each Latvian coastal sector occupies a distinct position within the Baltic sediment transport system:

- Liepaja—longshore polishing and sediment recycling.
- Ventspils—active erosion and continuous sediment replenishment.
- Riga—sediment retention and low-energy deposition.
- Salacgrīva—estuarine mixing with biological conditioning.

**Filtration and engineering applications.**

However, no direct filtration-performance experiments were conducted in this study; therefore, the application-oriented interpretations presented below are hypothesis-generating and intended to inform future adsorption and hydraulic testing. Surface chemistry is widely recognised as a key factor controlling adsorption behaviour. Rounded grains with abrasion-freshened surfaces (Liepaja) behave differently from:

- biologically coated grains (Salacgrīva),
- angular fractured grains (Ventspils),
- grains with hydrated particulate films (Riga).

These contrasts open pathways for evaluating natural sands for water-filtration and engineering applications, including ballast-water treatment systems.

## 5. Conclusions and Future Perspectives

This study provides a comprehensive analysis of beach sand samples collected from four coastal regions of Latvia. Using granulometric analysis, scanning electron microscopy (SEM), and X-ray photoelectron spectroscopy (XPS), we characterised the morphological, surface, and chemical properties of quartz sand grains.

Grain roundness, surface microtextural features, and surface chemistry proved to be critical indicators of sediment provenance, transport history, and environmental modification. The results reveal clear and systematic differences in grain morphology and surface coatings among sampling locations, reflecting contrasting hydrodynamic regimes, sediment supply, and geomorphological settings. These differences also allow identification of sand fractions with the greatest potential for applied technological use.

The integration of physical and surface-chemical data provides new insights into the natural evolution of Baltic coastal sands and establishes a scientific basis for selecting suitable raw materials for environmentally oriented technologies.

Furthermore, the results contribute to defining material-selection criteria that may inform future modification strategies aimed at improving the adsorption capacity of quartz sands in environmental remediation applications.

The present work represents a preliminary surface and microtextural characterisation; no direct adsorption, permeability, porosity, BET surface area, zeta potential, or hydraulic-performance measurements were performed.

The outcomes of this study therefore provide a materials-based foundation for subsequent applied development, including the functionalisation of selected quartz sands to enhance their sorption capacity for heavy metal ions (particularly Cr and Ni) and their potential application in ballast-water filtration systems. These aspects are considered perspectives for future research rather than results of the present study. Future work will focus on controlled surface modification approaches, such as UV irradiation or high-energy electron-beam exposure, to induce surface charging and optimise electrostatic interactions with target pollutants.

Future research directions include:

- Selecting sands with optimal morphology and surface chemistry, as identified in this study, for charge-functionalisation experiments.
- Investigating the influence of surface structure on charge retention and heavy-metal adsorption capacity.
- Developing and testing prototype filtration systems using functionalised Baltic sands as filter media.
- Assessing long-term charge stability, reusability, and environmental compatibility of treated sands.

This research pathway links fundamental sedimentological analysis with applied materials science and supports the development of sustainable, locally sourced filtration solutions for marine environmental protection.

## Figures and Tables

**Table 1:** Interpretation of surface microtextures in beach sand grains and their indicative transport environments based on visual roundness classes.

Roundness Class (Visual)	Diagnostic Microtextural Indicators (SEM)	Typical Processes/ Environments
Very angular	Fresh conchoidal fractures Sharp, high-relief edges Irregular shattering patterns Glacial crushing marks	Glacial grinding and crushing High-energy mechanical breakage Very short transport distance
Angular	Partially abraded edges Initial smoothing at asperities Micro steps and incipient solution pits Limited rounding of corners	Low–moderate energy fluvial transport Coastal reworking Glacial–fluvial mixing
Sub-angular	Uniform mechanical abrasion Low-relief grain surfaces Well-developed smoothing Mechanical impact pits; shallow solution basins	Moderate–long fluvial transport Wave action and littoral reworking Prolonged transport in beach/nearshore settings
Sub-rounded	Highly uniform abrasion patterns Extensive smoothing of edges Chemical micro-etching, low relief	Longshore drift Coastal dune reworking Multiple sediment recycling cycles Prolonged reworking in beach or nearshore environments
Rounded to well-rounded	Smooth, near-perfect grain outline Aeolian polish and frosting Strong chemical dissolution rounding Very low surface relief	Aeolian transport Mature dune systems Long-term abrasion in high-energy beach settings

**Table 2:** Microtextural features observed in SEM images and their environmental interpretation.

Microtextural Feature	Diagnostic Indicators (SEM)	Dominant Process	Liepaja	Riga	Ventspils	Salacgrīva
Angular grain surfaces	Sharp edges; planar fracture facets; lack of polish	Short fluvial transport; fresh detrital fragments	✔	✔✔✔		✔✔
Sub-angular surfaces	Partial rounding; residual fracture facets	Short to intermediate transport	✔	✔	✔✔	✔✔
Sub-rounded surfaces	Moderate abrasion; shallow pits; patchy polish	Littoral reworking medium-energy waves	✔✔		✔✔✔	✔✔✔
Rounded surfaces	Strong abrasion; smooth polish; very low relief	Intense surf-zone reworking; long transport	✔✔✔		✔✔	✔
Percussion pits	Circular to elliptical impact pits; frequent overlap	High-energy grain–grain collisions	✔✔✔		✔✔✔	✔✔
Microfractures/microchipping	Step fractures; scalloped edges	Mechanical brittle breakage	✔✔	✔✔	✔	✔✔

Notes: ✔ = present, ✔✔ = common, and ✔✔✔ = dominant.

**Table 3:** Detected ATR-FT-IR features and presence by sampling location (500–1800 cm^−1^ and 2500–4000 cm^−1^).

Wavenumber (cm^−1^)	Type	Liepaja	Riga	Salacgrīva	Ventspils	Most Plausible Assignment(s) in Sand Mixtures
533–534	shoulder	✔	✔	✔	✔	Framework silicate/feldspar low-wavenumber lattice/deformation modes [42].
591–594	shoulder	✔	✔		✔	Feldspar lattice/deformation region; bands in the ~584–604 cm^−1^ interval are reported for feldspar spectra in mineral-mixture IR contexts [42,43].
638–642	shoulder	✔	✔	✔	✔	Feldspar/aluminosilicate lattice/bending region near ~640–650 cm^−1^ reported in feldspar ATR-FTIR discrimination studies [43,44].
684.6	peak	✔	✔	✔	✔	Diagnostic quartz absorption region near 690–700 cm^−1^ (commonly around ~695 cm^−1^); apparent maxima may shift in mixtures and under ATR conditions due to band overlap [42,44,45,46].
725–750 *	shoulder	✔	✔	✔	✔	Overlap zone: carbonate ν_4_ neighborhood (~712 cm^−1^ calcite; ~728–730 cm^−1^ dolomite/aragonite) and possible feldspar contributions near ~720–740 cm^−1^; non-unique in mixtures [42,44,47,48,49]
764–766	peak	✔	✔	✔	✔	Quartz–feldspar fingerprint region; compatible with overlap between quartz bands (~779–798 cm^−1^ system) and feldspar contributions in mixed sands [42,44,45,46,47].
785–789	shoulder		✔	✔		Quartz diagnostic neighborhood (commonly discussed near ~798 and ~779 cm^−1^); may appear as a shoulder rather than a resolved doublet in mixtures/ATR [42].
871–875	shoulder	✔		✔	✔	Carbonate ν_2_ (CO_3_^2−^ out-of-plane bend), consistent with trace calcite/dolomite-type carbonates [45,48,49].
908.5	shoulder		✔			Clay/aluminosilicate contribution in the ~910–920 cm^−1^ region (Al–OH deformation/coupled modes depending on clay family and mixture) [44].
910–930 *	shoulder	✔	✔	✔	✔	Broad clay/aluminosilicate shoulder region widely reported around ~915–925 cm^−1^ in mineral-mixture IR and clay IR studies; often manifests as a shoulder in quartz–feldspar matrices [44].
971–983	peak	✔	✔	✔	✔	Sub-structure within the broad Si–O stretching envelope (framework silicates). Typically, not a stand-alone quartz identifier in mixtures [44].
1044–1047	shoulder	✔	✔	✔	✔	Mixed silicate Si–O stretching overlap domain (feldspar/clay contributions commonly populate ~1050–1000 cm^−1^) [42,43].
1090–1110 *	shoulder	✔	✔	✔	✔	High-intensity portion of the framework silicate Si–O stretching envelope (quartz/feldspar overlap; commonly discussed near ~1080–1100 cm^−1^) [43,44].
1129–1136	peak	✔	✔	✔	✔	Feldspar-associated Si–O stretching region; feldspar/quartz ATR-FTIR discrimination studies report feldspar bands in the ~1130–1150 cm^−1^ neighborhood, overlapping the high-wavenumber flank of quartz Si–O stretch in mixtures [43,46,47].
1165–1167	shoulder			✔		High-wavenumber Si–O stretching contribution reported for silica/quartz contexts near ~1160–1170 cm^−1^; may appear as a shoulder in mixtures [44].
1450.5	peak	✔		✔ *		Carbonate ν_3_ (CO_3_^2−^ asymmetric stretch) region; compatible with calcite/dolomite-type carbonates when accompanied by ν_2_ (~875 cm^−1^) [48,49].
2638.5	peak	✔	✔	✔	✔	Weak feature: carbonates exhibit combination/overtone bands in the ~2500–2600 cm^−1^ region in some reports, but the observed maximum at 2638.5 cm^−1^ is not a common primary diagnostic position for major sand minerals and is best treated as non-diagnostic without corroboration [42].
3735.3	peak		✔	✔		Weak OH stretch consistent with isolated/weakly H-bonded silanol (Si–OH) reported near ~3745–3750 cm^−1^ on silica/silicate surfaces [49,50,51].

* Indicates visually discernible but not detected by the automated peak-finder (per note in the provided summary table).

## Data Availability

The original contributions presented in this study are included in the article. Further inquiries can be directed to the corresponding author.

## Abbreviations

Cr	Chromium
EPS	Extracellular Polymeric Substances
SEM	Scanning Electron Microscopy
XPS	X-ray Photoelectron Spectroscopy
